# 4D-Flow Cardiac Magnetic Resonance Imaging: An 8-Year Clinical Practice Review

**DOI:** 10.31083/RCM46999

**Published:** 2026-01-09

**Authors:** Javier Urmeneta Ulloa, Vicente Martínez de Vega, Isabel Molina Borao, Ana Álvarez Vázquez, Julia López Alcolea, Manuel Recio Rodríguez, José Ángel Cabrera

**Affiliations:** ^1^Cardiology Department, Hospital Universitario Quirónsalud Madrid, 28223 Madrid, Spain; ^2^Radiology Department, Hospital Universitario Quirónsalud Madrid, 28223 Madrid, Spain; ^3^Faculty of Biomedical and Health Sciences, Universidad Europea de Madrid, 28670 Madrid, Spain; ^4^Pediatric Cardiology Department, Hospital Universitario Ramón y Cajal, 28034 Madrid, Spain

**Keywords:** 4D-flow, cardiac magnetic resonance, congenital heart disease, valvular heart disease, aortic disorders

## Abstract

Four-dimensional (4D) flow cardiac magnetic resonance (CMR) is an advanced imaging modality that enables comprehensive qualitative and quantitative assessment of blood flow in the three spatial dimensions plus time. This technique is more accurate, reproducible, and easier to interpret visually than conventional two-dimensional phase-contrast techniques. In this narrative review, we synthesize our clinical experience—including practical insights from representative cases from routine practice—with published research to describe the technical foundations, clinical applications, advantages, and limitations of this technique. We discuss the technical aspects, including spatial and temporal resolution, velocity encoding, contrast administration, workflow requirements, and post-processing software, and their influence on diagnostic performance. Thus, 4D-flow CMR imaging can accurately assess cardiac shunts through advanced visualization of pathlines and streamlines, providing direct quantification of pulmonary flow: systemic flow ratios, blood flow volumes, and complex hemodynamic patterns in congenital heart disease (CHD). Moreover, 4D-flow CMR imaging provides robust characterization of valvular and aortic disease through dynamic flow analysis and quantitative hemodynamic metrics. Overall, 4D flow CMR imaging is a powerful, noninvasive diagnostic tool that can greatly enhance clinical decision-making. The growing body of evidence supports the use of 4D-flow CMR imaging in routine clinical practice, particularly for evaluating CHD and valvular and aortic disorders.

## 1. Introduction

Four-dimensional (4D) flow cardiac magnetic resonance (CMR) imaging is an advanced technique that enables the comprehensive qualitative and quantitative assessment of intra- and extracardiac blood flow. This technique assesses four dimensions—the three spatial dimensions plus time—across the entire cardiac cycle with a single volumetric acquisition [1]. Building on conventional two-dimensional phase-contrast (2D-PC) magnetic resonance imaging (MRI), which has long been used to quantify cardiac blood flow [2, 3], 4D-flow CMR has proven valuable across a wide range of cardiac conditions. Moreover, this advanced imaging technique has numerous advantages over conventional techniques, especially in the assessment of congenital heart disease (CHD), aortic disorders, and valvular disease [4, 5, 6, 7].

In the present review, we summarize and discuss the essential principles required for the clinical implementation of 4D-flow CMR. In addition, we discuss the advantages of this technique as a tool to assess cardiovascular disease based on our clinical experience and on the published literature.

## 2. Technical Principles of 4D-Flow CMR

### 2.1 Acquisition and Image Analysis

Acquisition of the 4D-flow sequence is relatively straightforward. The first step is to define the volumetric coverage, which typically encompasses the heart and great vessels. A practical rule is that the acquisition volume should be “as large as necessary and as small as possible” [8]. Adequate box shimming should also be applied to minimize magnetic field inhomogeneities.

High spatial resolution is crucial to detect subtle flow phenomena and to ensure that the measurements are reproducible. However, excessive spatial resolution increases scan time and reduces the signal-to-noise ratio. An initial isotropic spatial resolution ≥2.5 mm^3^ is recommended, as the resolution usually decreases by nearly 50% after reconstruction. Temporal resolution should be as high as possible, with optimal values ranging from 40–50 ms [8, 9, 10].

The velocity encoding parameter (VENC) plays a critical role as it defines the maximum measurable velocity. The VENC setting is important because undersetting this parameter can lead to aliasing, while oversetting increases noise, degrades image quality, and underestimates true flow. In clinical practice, most authors recommend a VENC that is approximately 10% above the expected peak velocity [9, 11].

Although 4D-flow CMR can be performed without contrast agents, gadolinium improves the signal-to-noise ratio and vessel-to-tissue contrast. Based on our clinical experience, we recommend a slow, continuous infusion of gadolinium (0.1 mL/s) followed by a saline flush administered at the same rate. This approach ensures homogeneous distribution of the contrast agent and markedly improves image quality (both qualitatively and quantitatively) [12].

The most commonly cited limitation of 4D-flow CMR is the long acquisition time, which typically ranges from 7 to 10 minutes. Despite this drawback, the diagnostic benefits support the use of this technique in routine clinical practice [13, 14]. Importantly, ongoing advances in acceleration methods—including artificial intelligence (AI)–based reconstruction and k-space oversampling strategies (e.g., Sonic DL, SmartSpeed, Deep Resolve Boost, AiCE)—are expected to significantly reduce acquisition times in the near future.

After data acquisition, the raw images are transferred to institutional networks and processed with dedicated post-processing software. These tools enable quantification of flow velocity, volume, direction (via pathlines and streamlines), pressure gradients, and even advanced hemodynamic parameters such as wall shear stress (WSS) [15, 16]. When combined with three-dimensional (3D) qualitative analysis, post-processing analysis provides a comprehensive hemodynamic assessment of intracardiac and vascular flow, which is not possible with conventional cardiac MRI sequences.

### 2.2 Clinical Advantages of 4D-Flow CMR Versus 2D Phase-Contrast MRI

4D-flow CMR has multiple advantages over 2D-PC MRI, which has long been the standard tool for quantifying flow in cardiac MRI [2, 3]. First, the 3D volumetric acquisition of 4D-flow enables superior visual and quantitative assessment of hemodynamic phenomena [17, 18]. By contrast, 2D-PC MRI is limited to a single 2D slice displayed in grayscale; moreover, it is not possible to retrospectively reconstruct additional planes after acquisition.

With 4D-flow CMR, multiplanar reconstruction allows for the retrospective reorientation of the analysis plane, ensuring perpendicular alignment to the flow without the need to perform repeated image acquisition. This eliminates errors arising from suboptimal planning during the scan and markedly improves reproducibility [19]. In addition, blood flow can be tracked throughout the entire cardiac cycle, which means that the volumes across the valves, chambers, and intracardiac shunts can be directly and more accurately quantified than with 2D-PC MRI, which relies on indirect estimations [20, 21].

Another advantage of 4D-flow CMR is the capacity to quantify multiple structures simultaneously based on a single volumetric dataset. Whereas 2D-PC MRI requires multiple flow planes—thus requiring long scan times and a greater risk of error—4D-flow CMR provides all necessary measurements retrospectively from the same dataset [22, 23].

A final clinical advantage is that the integration of quantitative data with dynamic visualization (pathlines and streamlines) facilitates interpretation of complex flow patterns. This is particularly valuable in CHD, valvular disease, and aortic disorders, where a comprehensive understanding of hemodynamics is critical for clinical decision-making [3, 6, 9, 24].

## 3. Clinical Applications of 4D-Flow CMR

Although 4D-flow CMR has a wide range of clinical applications in cardiology, we have found it to be particularly valuable in the evaluation of CHD, valvular disorders, and aortic disease. However, this technique is beneficial in any clinical scenario requiring an accurate assessment of intra- or extracardiac flow [4, 5, 6, 9].

### 3.1 CHD and 4D-Flow CMR

The main clinical application of 4D-flow CMR is the evaluation of CHD, where the qualitative and quantitative data can help to better characterize and thus better understand this inherently complex condition. In addition, 4D-flow CMR eliminates the need for to perform multiple 2D-PC acquisitions [4, 23]. The 3D volumetric dataset of the heart and great vessels—encoded in color and covering the full cardiac cycle—provides a clear picture of the status of those structures. Moreover, the images provided by 4D-flow CMR are of higher quality than those obtained with 2D-PC MRI and easier to interpret, even for clinicians without specialized training in interpreting cardiac MRI images in CHD.

#### 3.1.1 Cardiac Shunts

4D-flow CMR can effectively assess all types of shunts, ranging from common lesions such as atrial septal defects (ASD) to the more complex anomalies described below [5].

3.1.1.1 Ostium Secundum ASD (OS-ASD)

**Fig. 1.**
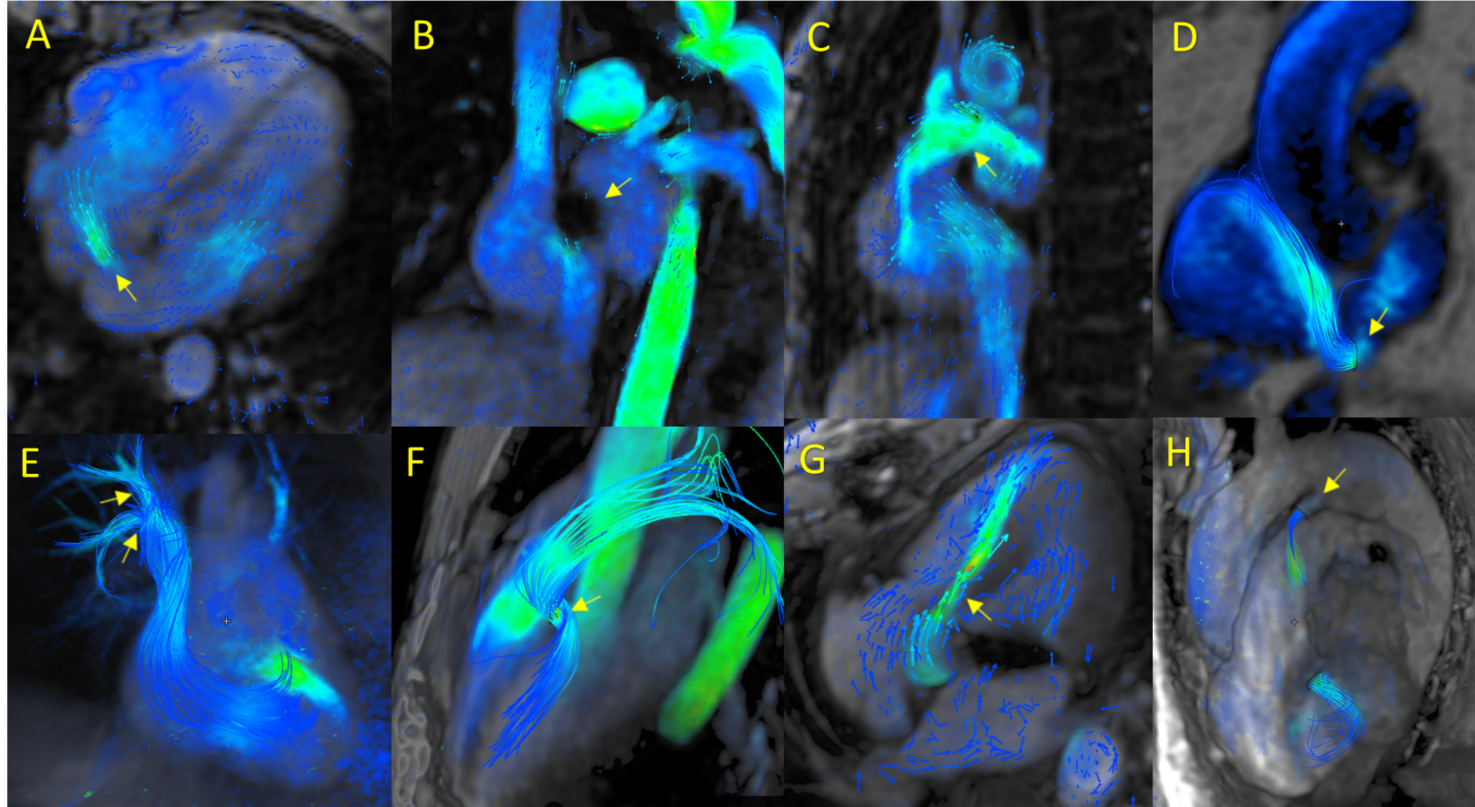
**4D-flow cardiac magnetic resonance (CMR) in cardiac shunts**. (A) Ostium secundum atrial septal defect (OS-ASD). (B) Absence of significant residual shunting following percutaneous OS-ASD closure. (C) Superior sinus venosus ASD. (D) Coronary sinus ASD. (E) Partial anomalous pulmonary venous return (PAPVR) involving the right upper and middle pulmonary veins draining into the superior vena cava. (F) Congenital perimembranous ventricular septal defect (VSD). (G) Iatrogenic VSD following prosthetic valve replacement. (H) Patent ductus arteriosus (PDA).

4D-flow CMR can be used to calculate pulmonary (Qp) and systemic (Qs) flows at any desired level and to directly quantify shunt volume. It also provides excellent visualization of the defect (Fig. 1A) to accurately determine the direction, size, and restrictive behavior (**Supplementary Video 1**). It also provides a comprehensive overview of hemodynamics in the right heart chambers and pulmonary arterial flow, thus facilitating clinical decision-making [5, 25]. It can also rule out the presence of significant residual shunts after percutaneous or surgical closure (Fig. 1B).

3.1.1.2 Non–OS-ASD

4D-flow CMR can readily identify ot her types of ASDs (apart from OS-ASD), including sinus venosus (Fig. 1C), primum ASD, and coronary sinus ASDs. 4D-flow CMR is especially valuable for detecting coronary sinus ASD (Fig. 1D), a rare condition that is often missed by conventional 2D-PC MRI. In such cases, a Qp:Qs mismatch may be observed on aortic or pulmonary 2D-PC MRI, even though pulmonary venous drainage to the left atrium appears normal and the interatrial septum remains intact. In these cases, a CT scan is normally needed to confirm the diagnosis. By contrast, 4D-flow CMR represents a major advance in the diagnosis of coronary sinus ASD, as the qualitative and quantitative data permits rapid detection through volumetric visualization, direct quantification of shunt volume, and accurate determination of the Qp:Qs ratio [26].

3.1.1.3 Partial Anomalous Pulmonary Venous Return (PAPVR)

4D-flow CMR has proven useful in the diagnosis of PAPVR. The multiplanar reconstructions and detailed 3D volumetric analysis facilitate the initial assessment of this condition (Fig. 1E). Shunt volume can be accurately and direct quantified through flow tracking at the site of the anomalous venous drainage. The combination of 4D-flow imaging with conventional determination of the Qp:Qs ratio yields an objective measure of hemodynamic significance [27] and also provides a comprehensive evaluation of all pulmonary veins to confirm their physiological connection to the left atrium (**Supplementary Video 2**). 4D-flow CMR is also highly valuable after surgical repair, particularly when contrast-related artifacts impede interpretation of the CT scan [28].

3.1.1.4 Ventricular Septal Defect (VSD)

4D-flow CMR plays an important role in evaluating ventricular septal defects to precisely determine the direction, size, and type (perimembranous, muscular, etc.) of shunt. In addition, it is easy to directly quantify the shunt volume and to calculate the Qp:Qs ratio. Importantly, it can be used to evaluate congenital VSDs (Fig. 1F) as well as postoperative iatrogenic defects (Fig. 1G, **Supplementary Video 3**) and ischemic ruptures in myocardial infarction (**Supplementary Video 4**).

3.1.1.5 Patent Ductus Arteriosus (PDA)

4D-flow CMR provides a detailed characterization of ductal morphology, length, and proximal/distal orifices (Fig. 1H, **Supplementary Video 5**). Similar to its role in other shunt types, 4D-flow CMR can rapidly, and with a high degree of reproducibility, determine shunt direction, flow volume, and the Qp:Qs ratio [23].

#### 3.1.2 Complex CHDs

3.1.2.1 Transposition of the Great Arteries (TGA)

**Fig. 2.**
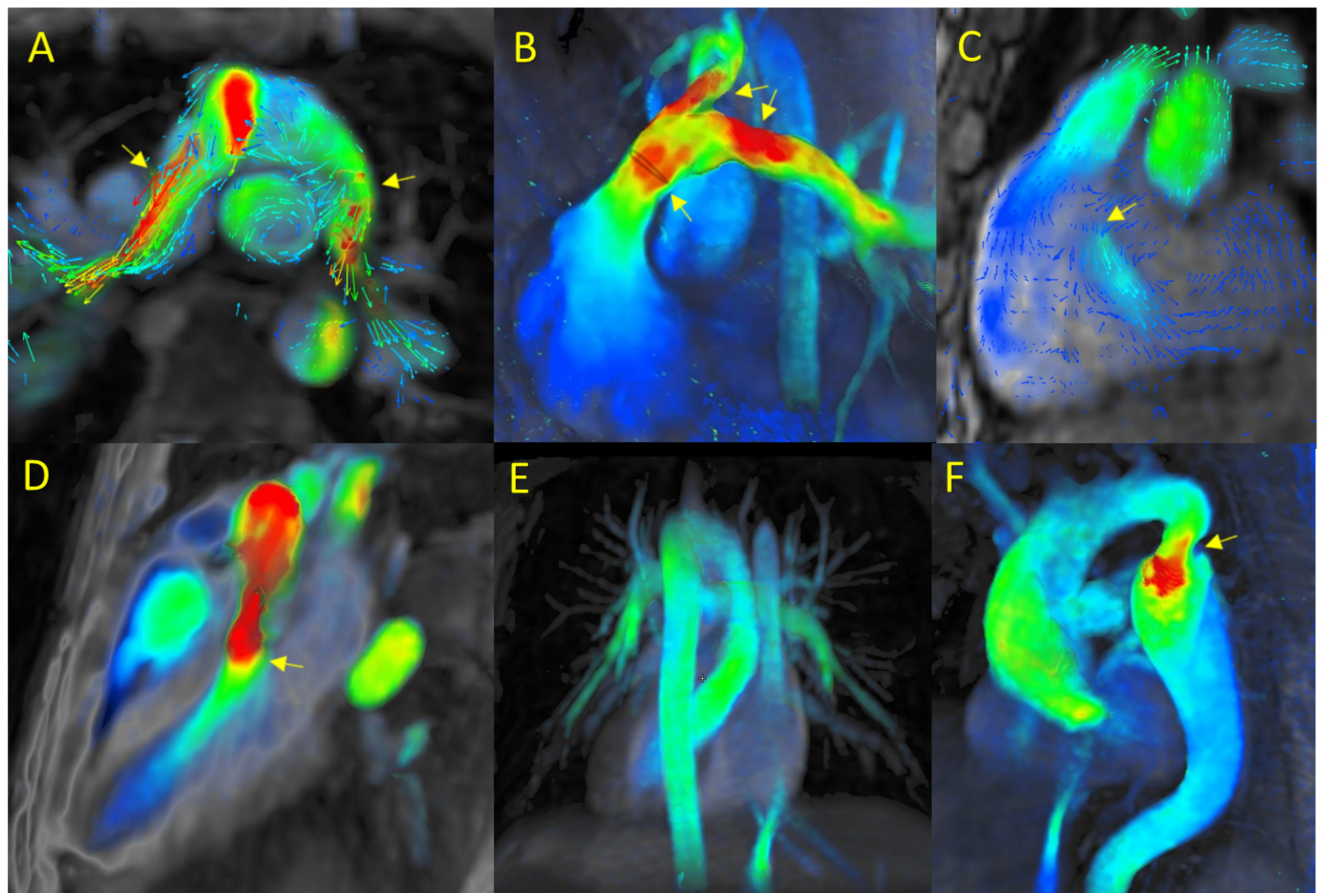
**4D-flow CMR in complex CHD**. (A) D-transposition of the great arteries (D-TGA) showing arterial status following anatomical repair (“Lecompte” maneuver). (B) Tetralogy of Fallot (TOF). In a patient with non-significant residual stenosis, flow acceleration is evident in the main pulmonary artery and its branches. (C) Ebstein anomaly. Tricuspid regurgitation with atrialization of the right ventricle. (D) Subaortic stenosis. Presence of accelerated and turbulent flow patterns in the subvalvular region. (E) Situs inversus. (F) Aortic coarctation.

In patients with more complex anatomy, such as D-transposition of the great arteries (D-TGA), 4D-flow CMR can qualitatively and quantitatively assess the patient’s status after physiologic correction. In addition, 4D-flow imaging can rule out pulmonary venous or caval vein stenoses and baffle dehiscence. It provides multiplanar visualization of the parallel arrangement of the great arteries (**Supplementary Video 6**) to detect the presence of associated dilatation or valvular disease. In cases of regurgitation, atrioventricular valve competence can be assessed and quantified. After anatomical correction with the “Lecompte” maneuver (Fig. 2A), multiplanar reconstructions provide valuable data to assess how the surgical repair functions, and to detect the presence of branch pulmonary artery stenosis.

3.1.2.2 Tetralogy of Fallot (TOF)

The main benefit of 4D-flow CMR in TOF (Fig. 2B) is the ability to simultaneously and noninvasively evaluate cardiac and vascular hemodynamics across multiple planes. Unlike conventional imaging methods, 4D-flow CMR can accurately quantify pulmonary regurgitation, characterize abnormal flow patterns, and estimate cardiac output distribution across pulmonary branches in a single volumetric acquisition (**Supplementary Video 7**). In turn, these data can be used to assess right ventricular remodeling and the functional consequences of the outflow tract. This is especially useful for long-term follow-up and personalized therapeutic planning [4, 23].

3.1.2.3 Ebstein Anomaly

4D-flow CMR can directly quantify tricuspid regurgitation by tracking the regurgitant jet, thus making it possible to calculate the regurgitant fraction (RF), a key parameter—together with right ventricular volume and atrialized portion of the right ventricle—for clinical management (Fig. 2C). The characteristic apical displacement of the septal tricuspid leaflet can also be readily identified [29, 30] (**Supplementary Video 8**).

3.1.2.4 Subaortic Stenosis

4D-flow CMR provides a detailed characterization of accelerated and turbulent flow patterns in the subvalvular region in subaortic stenosis (Fig. 2D), which yields a more accurate estimate of the pressure gradient than conventional methods. In addition, high-velocity jets can be visualized in 3D relative to left ventricular outflow tract geometry, thus providing a better assessment of the hemodynamic impact of obstruction (**Supplementary Video 9**), which is useful to monitor progression of the lesion and to determine the optimal timing of surgical intervention.

3.1.2.5 Situs Inversus

4D-flow CMR provides a comprehensive assessment of the anatomy and hemodynamics in patients with situs inversus (Fig. 2E). Flow patterns in the heart chambers and great vessels can be visualized in 3D to provide a better understanding of atrioventricular and ventriculoarterial connections and any associated abnormalities (e.g., obstructions, regurgitations, or shunts). 4D-flow CMR provides valuable data needed to make the initial diagnosis and for follow-up. It also provides the all relevant data needed to plan the surgical intervention in patients with complex CHD (**Supplementary Video 10**).

3.1.2.6 Aortic Coarctation (CoA)

Aortic coarctation is another congenital condition in which 4D-flow CMR has proven its utility (Fig. 2F). Compared with conventional 2D imaging, which requires sequential quantification at multiple sites (including the aortic valve, sinotubular junction, proximal ascending aorta, and descending thoracic aorta), 4D-flow CMR is much more efficient, allowing for all relevant data to be acquired in a single study (**Supplementary Video 11**). The visualization capabilities of this technique are helpful to determine (or rule out) the presence of coarctation. In some cases, it may also be useful to detect the presence of hemodynamically-relevant collateral vessels. The same volumetric dataset can be used to evaluate intracardiac shunts and concomitant valvular disease [31, 32].

### 3.2 Valvular Heart Disease (VHD) and 4D-Flow CMR

4D-flow CMR is particularly useful to quantify valvular regurgitation in the semilunar and atrioventricular valves in VHD [7, 12, 31].

#### 3.2.1 Semilunar Valves 

4D-flow CMR has emerged as an important tool to evaluate hemodynamics in the aorta and pulmonary artery, where its ability to acquire complete 3D volumetric datasets over the entire cardiac cycle allows for repeated measurements at multiple vascular sites without the need to reposition acquisition planes, thereby ensuring greater reproducibility and accuracy (**Supplementary Video 12**). 4D-flow CMR is more reliable than conventional 2D techniques in quantifying the regurgitant volume and RF. It also provides insights into complex flow patterns—including vortices, eccentric jets, and turbulence—that may influence both quantification and disease progression (**Supplementary Video 13**). These data can be used to better characterize the hemodynamic burden of aortic and pulmonary regurgitation to obtain a more accurate determination of severity, which can then be used to create a highly-individualized treatment plan [33].

**Fig. 3.**
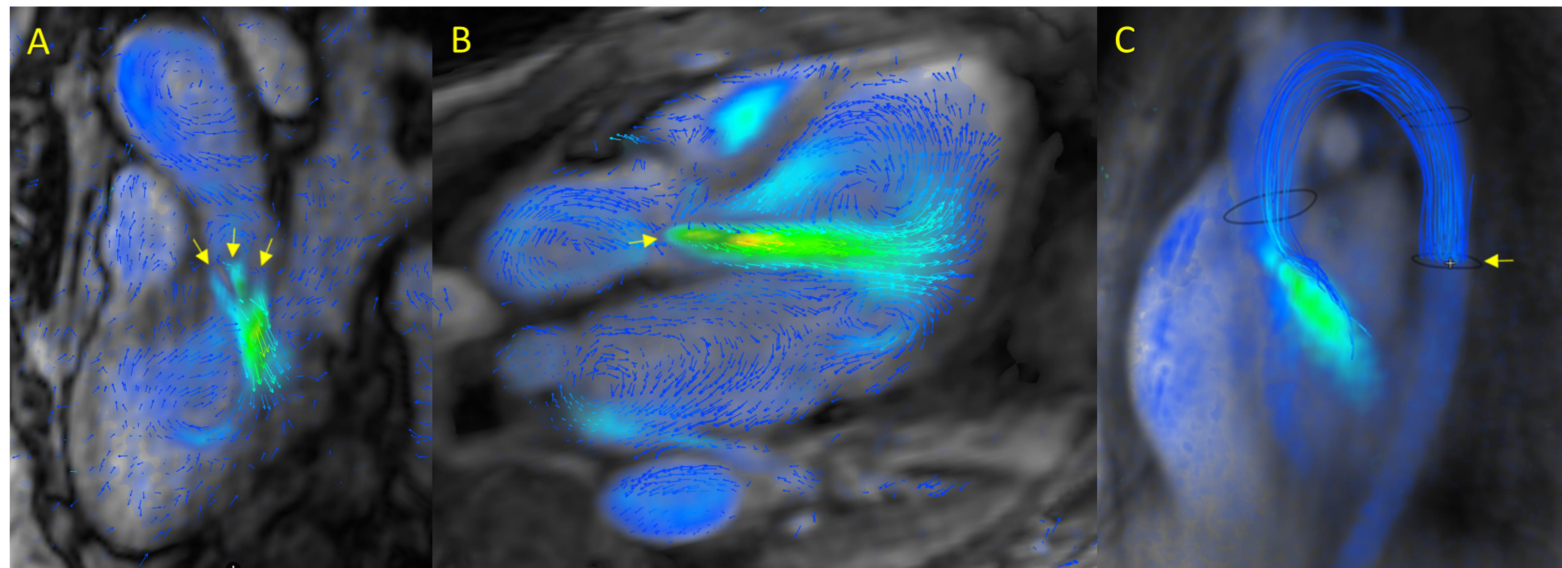
**4D-flow CMR in valvular heart disease (VHD): semilunar valves**. (A) Aortic regurgitation with three separate regurgitant jets. (B) Severe aortic regurgitation. (C) Holodiastolic and significant flow reversal in the descending thoracic aorta in a patient with severe aortic regurgitation.

Flow patterns are commonly measured at the sinotubular junction where flow is less variable in order to quantify the RF [34], with severe regurgitation (RF ≥30%) being common in aortic regurgitation (Fig. 3A–C). 4D-flow CMR makes it easy to visually identify these complex flow patterns, particularly in patients with aortic stenosis, bicuspid valve, or aortic dilatation. In this setting, it is crucial to assess flow in the descending thoracic aorta (Fig. 3C, **Supplementary Video 14**) given that holodiastolic flow reversal combined with an RF ≥17% have been consistently associated with severe aortic regurgitation. Determination of flow provides an additional, highly valuable data point to ensure accurate grading of regurgitation severity [12]. In pulmonary regurgitation, 4D-flow CMR can determine flow patterns (regurgitant volume, RF, peak velocity, etc.) in both the main and branch pulmonary arteries. In turn, these data can be used to evaluate the impact on right ventricular flow and to rule out associated stenoses [9, 20].

#### 3.2.2 Atrioventricular Valves 

**Fig. 4.**
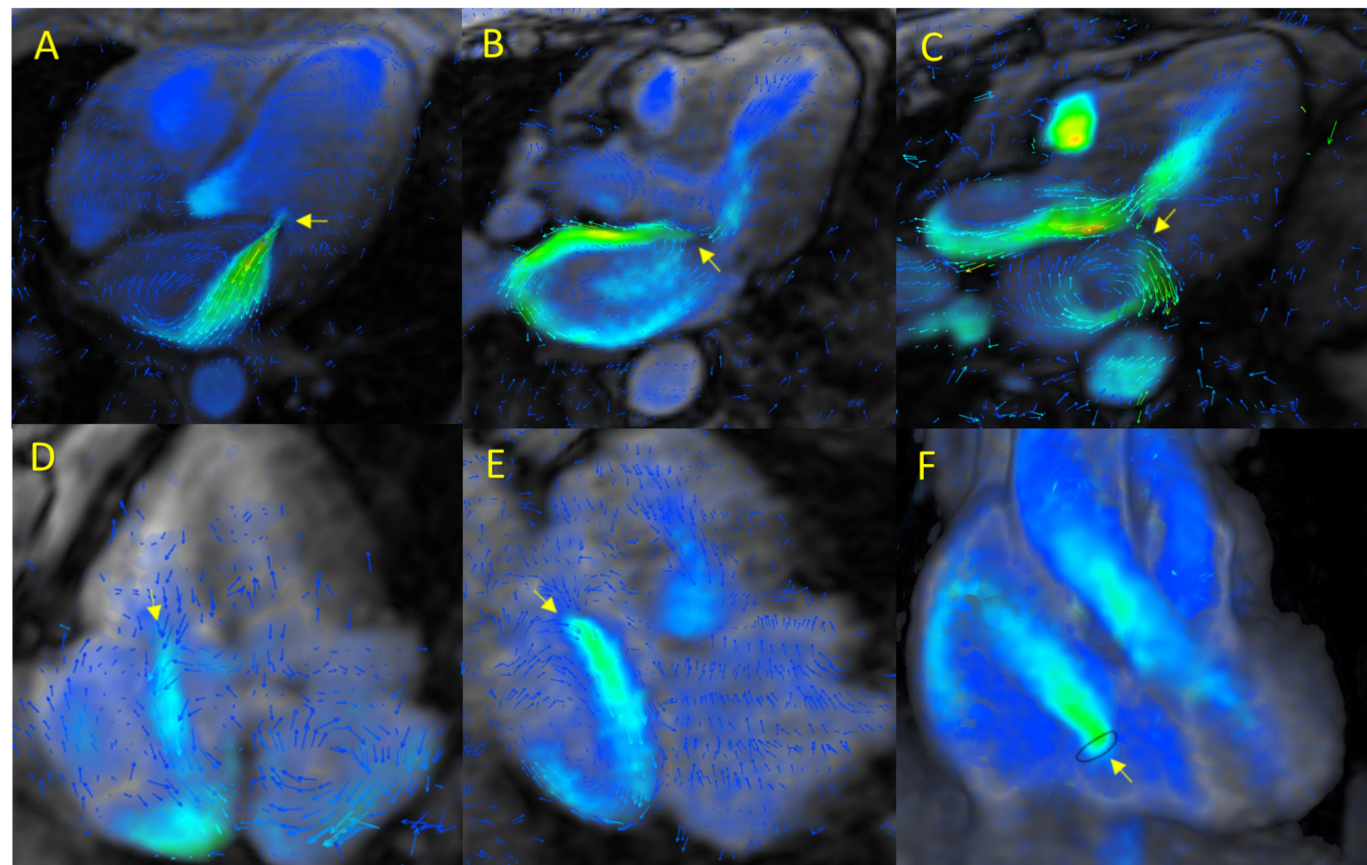
**4D-flow CMR in VHD: atrioventricular valves**. (A) Secondary (functional) mitral regurgitation due to mitral leaflet tenting. (B) Primary mitral regurgitation in the context of posterior mitral leaflet prolapse. (C) Mitral regurgitation associated with systolic anterior motion in a patient with hypertrophic obstructive cardiomyopathy. (D,E) Tricuspid regurgitation visualized with multiplanar reconstructions. (F) Tricuspid regurgitation assessed by volumetric reconstruction.

A major advantage of 4D-flow CMR in evaluating atrioventricular valves is its ability to directly quantify regurgitant volume and RF by tracking the regurgitant jet across the entire cardiac cycle at the valve plane [35]. The retrograde flow can be visualized in three dimensions (Fig. 4A–F, **Supplementary Videos 15–18**), even in the presence of multiple or eccentric jets, whose evaluation is more challenging when conventional imaging techniques are used and/or in the presence of intracardiac shunts (**Supplementary Video 19**).

An alternative methodological approach to determining regurgitation in the atrioventricular valves is to make an indirect calculation [36] based on the difference between forward flow through the atrioventricular valve and flow at the corresponding semilunar valve. This strategy correlates closely with standard 2D-PC MRI while avoiding the complexity involved in directly analyzing highly turbulent jets. Nevertheless, based on our clinical experience and that of other groups [37], we believe that direct jet quantification is more precise and reliable in clinical practice, as it avoids the need to rely on geometric assumptions and also minimizes cumulative measurement errors. That said, the indirect method may be a useful complementary approach in certain well-defined clinical scenarios, where it may even be more reproducible. Ultimately, more evidence is needed to determine the optimal approach to assessing mitral and tricuspid valve regurgitation with 4D-flow CMR, which may involve both indirect and direct methods.

Although 4D-flow CMR has limitations in grading valvular stenosis (**Supplementary Video 20**), it is the most robust approach to quantifying regurgitation, as all regurgitant lesions can be comprehensively evaluated based on a single volumetric acquisition. This is useful because it eliminates reliance on indirect estimates and provides an integrative assessment, which is difficult to achieve with conventional cardiac imaging methods.

### 3.3 Aortic Disorders and 4D-Flow CMR

4D-flow CMR has proven to be highly useful in evaluating aortic disorders. Not only has it improved our understanding of the pathophysiological mechanisms underlying these conditions, but is also highly useful for clinical follow-up [6, 16, 38]. It can characterize flow patterns and estimate WSS (**Supplementary Video 21**), thus helping to identify the hemodynamic mechanisms that promote vascular remodeling, drive aortic dilatation, and increase the risk of acute aortic events [39, 40].

#### 3.3.1 Chronic Aortic Dissection 

**Fig. 5.**
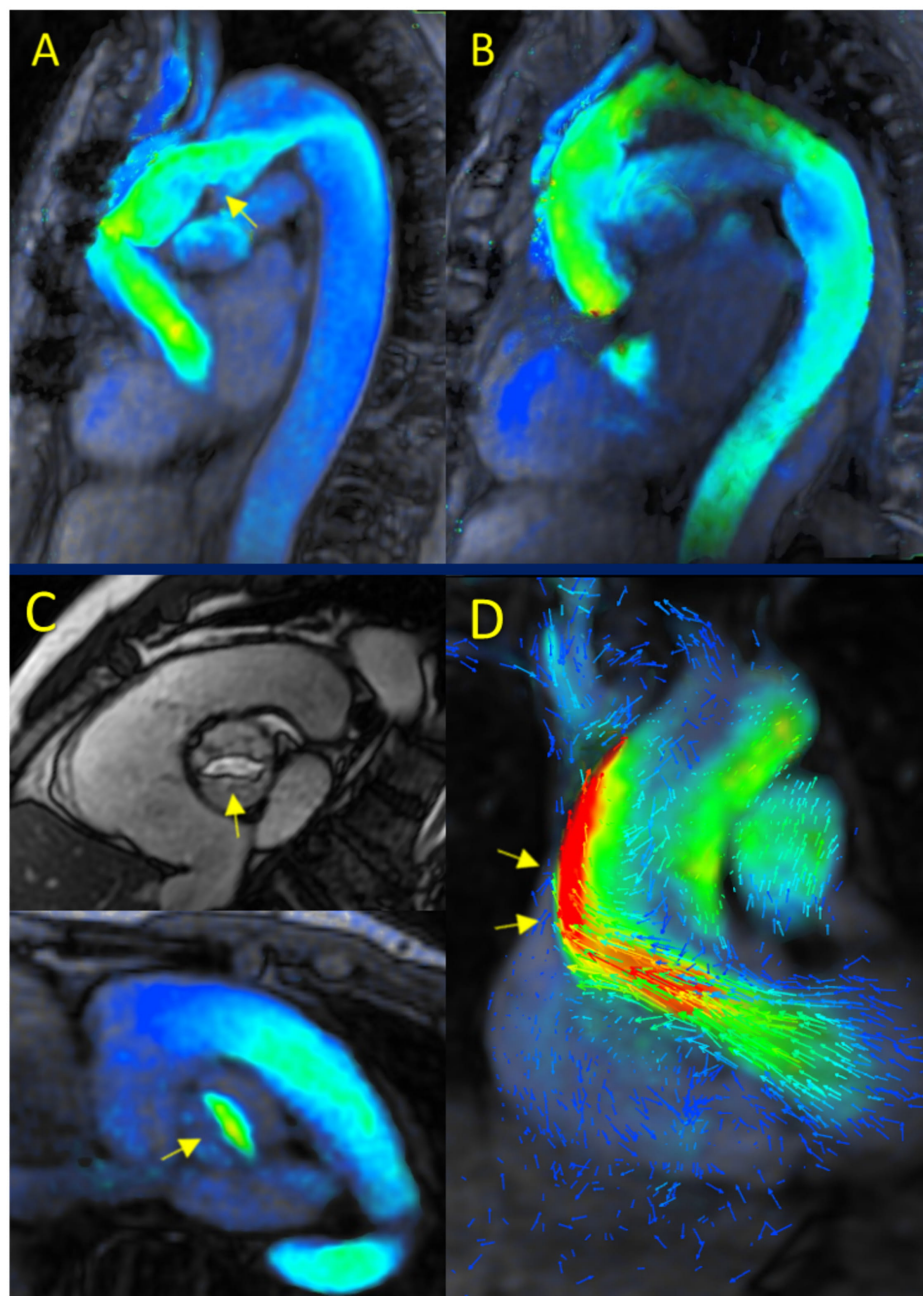
**4D-flow CMR in aortic disorders**. (A,B) Chronic aortic dissection. (A) Surgically treated aortic dissection with a pseudoaneurysm at the transition zone between the implanted aortic graft and the aortic arch. (B) Surgically treated aortic dissection with valved prosthetic graft in the ascending aorta and patent stent in the aortic arch–proximal descending thoracic aorta. (C,D) Patient with a bicuspid aortic valve, morphotype type 1 (right–left coronary cusp fusion) (C: Steady-state free precession (SSFP), top; 4D-flow CMR, bottom), with flow directed toward the anterior region of the aorta, leading to aortic dilation (D).

In this condition, 4D-flow CMR is complementary to computed tomography (CT). While CT scans provide high-resolution anatomic detail, 4D-flow CMR adds important functional data. It can also be used to monitor the outcomes of aortic dissection (Fig. 5A,B, **Supplementary Video 22**), which is important because it obviates the need to perform CT scans, thus greatly reducing radiation exposure. As our group has previously shown [6], 4D-flow CMR can accurately differentiate between true and false lumens, which is essential to properly interpret intraluminal hemodynamics. It also can be used to assess branch perfusion according to lumen dependency and residual flow within the false lumen. These parameters are critical for risk stratification, since persistent flow in the false lumen has been associated with progressive aortic enlargement and poorer prognosis. Detailed hemodynamic analysis further enhances treatment decision-making and plays a pivotal role in developing personalized strategies for follow-up.

#### 3.3.2 Aortic Dilatation and Bicuspid Aortic Valve (BAV) 

4D-flow CMR is particularly useful to assess these conditions (Fig. 5C,D) as it can characterize abnormal flow patterns such as helical flow, which have been closely linked to pathological remodeling of the ascending aorta. Quantification of WSS is important because persistently elevated WSS has been associated with progressive aortic dilatation and increased risk of complications, including dissection and rupture [39, 40, 41]. These findings support the hypothesis that, apart from anatomical factors, the hemodynamic environment plays a key role in the clinical evolution of patients with BAV.

4D-flow CMR is emerging as a promising risk stratification tool across the spectrum of aortic disease. Several recent studies have demonstrated that flow-derived parameters such as flow eccentricity, helical patterns, and regional WSS distribution [41, 42] correlate more strongly with disease progression than aortic diameter alone, which at present is the main criterion for surgical intervention. This is important given that a substantial proportion of aortic dissections are carried out in cases in which the aortic diameter is below conventional surgical thresholds. In patients with hereditary aortopathies such as Marfan or Loeys–Dietz syndrome, 4D-flow CMR can detect early hemodynamic alterations that may not be apparent on vascular morphology. The presence of these alterations could potentially be used as subclinical biomarkers of disease progression to better inform the therapeutic approach in these high-risk cohorts.

## 4. Application of 4D-Flow CMR in Technically Challenging Scenarios

**Fig. 6.**
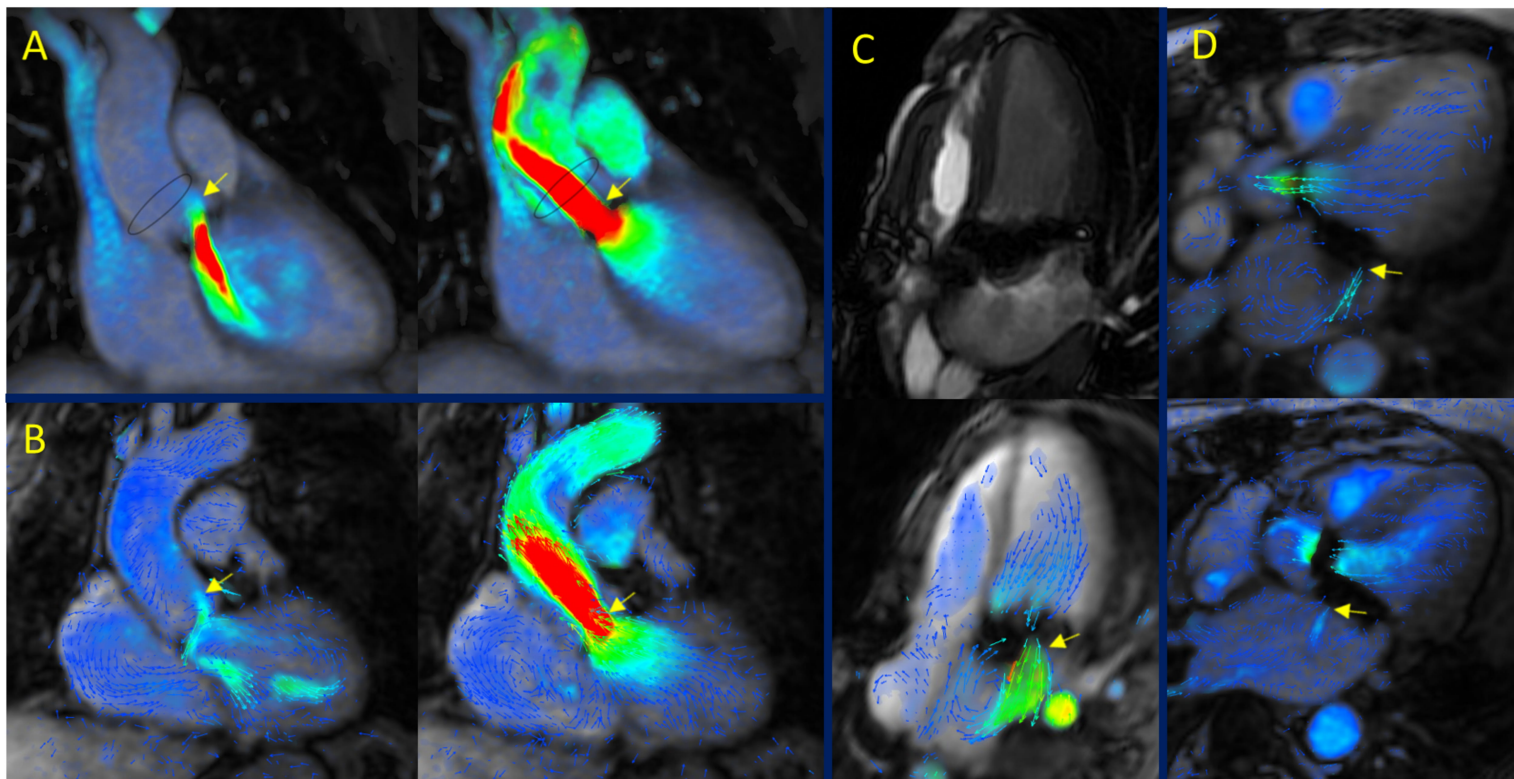
**4D-flow CMR in cardiac valve prostheses**. (A,B) Aortic bioprosthesis with prosthetic degeneration, demonstrating significant aortic regurgitation (left) and prosthetic stenosis (right). (C) Severe intra-annular regurgitation in a patient with mitral valve repair. SSFP cine imaging (top) there is no clear evidence of turbulent flow suggestive of mitral regurgitation; by contrast, on 4D-flow CMR (bottom), there is a prominent central intra-annular regurgitant jet. (D) Non-significant periprosthetic leaks in mitral mechanical prostheses (top and bottom).

**Fig. 7.**
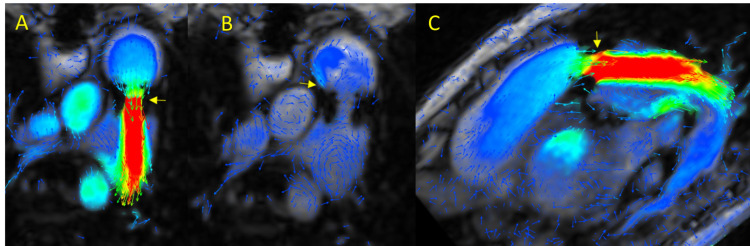
**4D-flow CMR in a pulmonary valve prosthesis**. (A) Flow acceleration secondary to pulmonary valve prosthesis stenosis (axial reconstruction); flow is preferentially directed toward the left pulmonary artery. (B) Mild prosthetic pulmonary regurgitation. (C) Flow acceleration secondary to pulmonary valve prosthesis stenosis (sagittal reconstruction).

4D-flow CMR has shown it value in complex clinical settings where conventional imaging is insufficient due to its inherent limitations. For example, in conventional 2D imaging, the metallic components of prosthetic heart valves generate magnetic field inhomogeneities that impair flow quantification. By contrast, with 4D-flow CMR, the 3D vector-based images of blood flow around these prostheses make it possible to evaluate residual gradients, paravalvular leaks, and abnormal hemodynamic patterns (Figs. 6A–D,7; **Supplementary Videos 23,24**) [43, 44].

In patients with implanted intracardiac devices (e.g., pacemaker or cardioverter defibrillators), magnetic susceptibility artifacts frequently compromise image quality in standard sequences [45, 46]. However, these artifacts can be largely mitigated by the use of 4D-flow acquisition and reconstruction strategies, as shown in *ex vivo* experiments [47] and in our own clinical experience (**Supplementary Video 25**). As a result, intracavitary and transvalvular flows can be reliably assessed with 4D-flow CMR, thus making this tool indispensable in this patient population.

4D-flow CMR is more robust than conventional techniques in patients with prostheses or intracardiac devices due to the type of sequences employed. Conventional cine MRI typically relies on steady-state free precession (SSFP) sequences, which provide excellent blood–myocardium contrast but are highly sensitive to magnetic field inhomogeneities, leading to banding artifacts and extensive signal loss in the presence of metallic hardware. By contrast, 4D-flow CMR is based on gradient-echo sequences, which are less vulnerable to variations in local susceptibility, thereby reducing the magnitude of artifacts caused by generators and leads [48]. Moreover, 4D-flow CMR has relatively short echo times and phase corrections, which limits spatial distortion and increases the accuracy of flow quantification. Unlike cine sequences, which rely primarily on signal intensity, 4D-flow CMR is based on velocity-encoded phase information, which provides clinically meaningful hemodynamic data even in regions where the magnitude signal is compromised. All of these features explain the superior robustness of 4D-flow CMR in patients with prosthetic valves and intracardiac devices, thus supporting its diagnostic value in these patients, particularly when compared to the limitations of conventional imaging techniques.

In atrial fibrillation (AF), 4D-flow CMR has numerous advantages over conventional 2D PC MRI. The irregular R-R intervals in AF make it challenging to acquire 2D images, which require stable electrocardiogram gating and multiple breath holds to obtain plane-specific measurements, thereby increasing the likelihood of artifacts and misalignment [1, 49]. By contrast, 4D-flow CMR is acquired during free breathing using self-gating and motion-correction techniques, which reduce susceptibility to respiratory and cardiac irregularities [50]. Furthermore, the integration of multiple cardiac cycles yields a more physiologic representation of atrial hemodynamics, thus minimizing biases introduced by beat-to-beat variability. Similarly, 4D-flow CMR provides complete volumetric coverage of the left atrium and the left atrial appendage, allowing clinicians to retrospectively evaluate any plane of interest. By contrast, 2D sequences require prospective slice planning, an important limitation.

The applications of 4D-flow CMR described above highlight the robust and versatile diagnostic capabilities of this technique, which provides quantitative data with advanced hemodynamic visualization in settings where the diagnostic accuracy of conventional imaging techniques is insufficient.

### Limitations of 4D-Flow CMR

Despite the numerous strengths of 4D-flow CMR, it also has certain limitations and challenges. Importantly, however, those limitations do not represent a true barrier to implementing this technique in clinical practice. One widely acknowledged limitation is the long acquisition time (7–10 minutes); although a shorter acquisition time would be desirable, in our clinical experience, this time range is acceptable and clinically cost-effective. Moreover, the recent development of AI–based acceleration methods [51], including k-space oversampling strategies, is expected to significantly reduce scan times in the near future.

Another potential limitation is the need to define an initial VENC, which may pose challenges for clinicians who have limited experience with 4D-flow CMR. In practice, however, this is rarely problematic, since the VENC can be retrospectively adjusted during post-processing, with priority given at acquisition to avoiding aliasing. Furthermore, the development of dual- and multi-VENC sequences is a promising solution to this potential difficulty, particularly in patients with coexisting stenotic and regurgitant lesions [52].

Spatial resolution is another potential limitation, as very small flow phenomena such as tiny shunts (e.g., atrial septal defects <5 mm) may go undetected. Nevertheless, such defects are not clinically relevant, as they do not change the prognosis or influence therapeutic decision-making. Moreover, our experience shows that 4D-flow CMR detects all shunts with a significant hemodynamic impact, a finding that has been confirmed by other authors [4, 5].

Another possible limitation of this technique is the quantification of valvular regurgitation. At present, the severity thresholds used in 4D-flow CMR are extrapolated from validated studies of 2D-PC MRI because no validated studies have been performed to date to establish these parameters for 4D-flow CMR.

An inherent limitation of 2D-PC MRI is the inability to reliably asses stenosis and determine pressure gradients. While 4D-flow CMR can provide complementary information, it faces the same limitations of conventional imaging.

The lack of standardization is another challenge for 4D-flow CMR. In this regard, there is a clear need to conduct multicenter studies to compare results across scanners and post-processing platforms from different manufacturers. At our center, we use a single commercial platform (GE Medical Systems; Milwaukee, WI, USA) with dedicated software (Tempus Pixel. Chicago, IL, USA), which may yield different results when compared to other scanner types in other settings.

A final but important limitation of 4D-flow CMR is the cost, which remains a key obstacle to more widespread adoption. Nevertheless, the costs are expected to decline over time, which should gradually increase its use in standard clinical workflows.

## 5. Conclusions

The development of 4D-flow CMR is undoubtedly one of the most significant advances in the evaluation of cardiovascular disease in recent years. The ability to obtain, in a single volumetric acquisition, both qualitative and quantitative information on blood flow in three spatial dimensions plus time is highly valuable in the clinical setting.

4D-flow CMR enables the comprehensive, intuitive analysis of most cardiac defects—both simple and complex—in patients with CHD, obviating the need to perform multiple 2D-PC acquisitions. In valvular heart disease, this technique enables the direct quantification of regurgitant volumes and fractions. Moreover, it is highly reproducible, thus overcoming on of the main limitations of indirect methods. In aortic disease, 4D-flow CMR provides a detailed characterization of flow patterns and their hemodynamic impact on the vascular wall, with relevant applications in the evaluation of dissection and dilatation.

Although 4D-flow CMR has some limitations—mainly acquisition time, the need for specialized post-processing, and a lack of standardization—advances in acceleration strategies—including AI—and a small but growing body of evidence support the value of implementing 4D-flow CMR in routine clinical practice

At our institution, we currently use 4D-flow CMR in approximately 60% of patients who require cardiac MRI. In the near future, the continued development of AI for use in acquisition and post-processing will likely lead to 4D-flow CMR becoming the standard of care.

Where available, 4D-flow CMR should be considered essential in patients with a confirmed or suspected diagnosis of CHD, valvular or aortic disorders, unexplained ventricular dilatation, and in technically challenging scenarios where conventional sequences are insufficient to accurately determine blood flow.

In summary, 4D-flow CMR is a highly valuable tool in routine cardiac imaging. In time, this technique is expected to become the reference standard in MRI for assessing cardiovascular hemodynamics due to its capacity to provide precise diagnostic data that are essential for selecting the optimal therapeutic approach for each patient.

## Supplementary Material

Supplementary material associated with this article can be found, in the online version, at https://doi.org/10.31083/RCM46999.
