# Supplementary material for: 4D-Flow Cardiac Magnetic Resonance Imaging: An 8-Year Clinical Practice Review
Source: Rev Cardiovasc Med. 2026 Jan 9;27(1):46999. doi: 10.31083/RCM46999 (PMC12873672; doi:10.31083/RCM46999)
Supplement: Supplementary file 1 [file 2153-8174-27-1-46999-s1.zip › Supplementary Videos.docx]

**Supplementary Video Legends**

**Supplementary Video 1.** 4D-flow CMR. Ostium secundum ASD (OS-ASD).

**Supplementary Video 2.** 4D-flow CMR showing a partial anomalous pulmonary venous return (PAPVR) involving the left superior pulmonary vein, with volumetric and multiplanar reconstructions illustrating the anomaly. At the end of the video, the remaining pulmonary veins are observed draining physiologically into the left atrium.

**Supplementary Video 3.** 4D-flow CMR. Perimembranous ventricular septal defect following prosthetic valve replacement.

**Supplementary Video 4.** 4D-flow CMR showing a muscular-type ventricular septal defect following acute myocardial infarction. A left-to-right shunt (evidenced by pathlines) is observed, with flow directed from the left to the right ventricle and subsequently into the pulmonary artery.

**Supplementary Video 5.** 4D-flow CMR. Patent ductus arteriosus (PDA)

**Supplementary Video 6.** 4D-flow CMR in a patient with D-Transposition of the great arteries (D-TGA) following physiologic correction (Senning procedure). Findings: parallel outflow of the great vessels (aorta and pulmonary artery) typical of this condition, without semilunar valvulopathy; aorta located anteriorly and to the right of the pulmonary valve; axial reconstruction illustrating preserved patency of pulmonary venous and caval pathways, without significant baffle compromise; and Qp:Qs quantification showing no significant residual shunts.

**Supplementary Video 7.** 4D-flow CMR. Tetralogy of Fallot (TOF). Severe “free” pulmonary regurgitation with mild residual branch pulmonary artery stenosis after childhood surgical repair.

**Supplementary Video 8.** 4D-flow CMR in Ebstein anomaly. Four-chamber and short-axis reconstruction showing atrialization of the right ventricle with no significant tricuspid regurgitation

**Supplementary Video 9.** 4D-flow CMR in subaortic stenosis. Three-chamber and coronal reconstruction.

**Supplementary Video 10.** 4D-flow CMR in situs inversus. No associated valvular disease or significant shunts.

**Supplementary Video 11.** 4D-flow CMR in aortic coarctation. Volumetric reconstruction enabling multiple measurements to evaluate hemodynamic significance from a single three-dimensional acquisition.

**Supplementary Video 12**. 4D-flow CMR in aortic regurgitation. Severe aortic regurgitation (regurgitant fraction = 46%), with measurements made at multiple levels along the aorta.

**Supplementary Video 13**. 4D-flow CMR in aortic regurgitation. Aortic regurgitation with three distinct regurgitant jets.

**Supplementary Video 14.** 4D-flow CMR in aortic regurgitation. Holodiastolic and significant flow reversal in the descending thoracic aorta observed in a patient with severe aortic regurgitation.

**Supplementary Video 15.** 4D-flow CMR in mitral regurgitation: assessment of primary MR due to posterior mitral leaflet prolapse.

**Supplementary Video 16.** 4D-flow CMR in mitral regurgitation. Mitral regurgitation in a patient with hypertrophic obstructive cardiomyopathy.

**Supplementary Video 17.** 4D-flow CMR in secondary mitral regurgitation.

**Supplementary Video 18.** 4D-flow CMR in tricuspid regurgitation.

**Supplementary Video 19**. 4D-flow CMR in patient with perimembranous ventricular septal defect and tricuspid regurgitation.

**Supplementary Video 20.** 4D-flow CMR in a patient with moderate valvular pulmonary stenosis.

**Supplementary Video 21.** 4D-flow CMR in aortic disorders: flow pattern characterization and wall shear stress assessment in a patient with a proximal descending thoracic aortic pseudoaneurysm.

**Supplementary Video 22.** 4D-flow CMR in chronic aortic dissection. Surgically treated aortic dissection, pseudoaneurysm at the transition zone between the implanted aortic graft and the aortic arch.

**Supplementary Video 23**. 4D-flow CMR in cardiac valve prostheses. Aortic bioprosthesis with prosthetic degeneration, demonstrating significant aortic regurgitation and prosthetic stenosis.

**Supplementary Video 24.** 4D-flow CMR in a patient with mitral valve repair and severe intra-annular regurgitation.

**Supplementary Video 25.** 4D-flow CMR in a patient with pacemaker implantation and mild aortic regurgitation.
